# ASSIST‐U: A system for segmentation and image style transfer for ureteroscopy

**DOI:** 10.1049/htl2.12065

**Published:** 2023-12-18

**Authors:** Daiwei Lu, Yifan Wu, Ayberk Acar, Xing Yao, Jie Ying Wu, Nicholas Kavoussi, Ipek Oguz

**Affiliations:** ^1^ Department of Computer Science Vanderbilt University Nashville Tennessee USA; ^2^ Department of Urology Vanderbilt University Medical Center Nashville Tennessee USA

**Keywords:** computerised tomography, computer vision, data visualisation, endoscopes, image segmentation

## Abstract

Kidney stones require surgical removal when they grow too large to be broken up externally or to pass on their own. Upper tract urothelial carcinoma is also sometimes treated endoscopically in a similar procedure. These surgeries are difficult, particularly for trainees who often miss tumours, stones or stone fragments, requiring re‐operation. Furthermore, there are no patient‐specific simulators to facilitate training or standardized visualization tools for ureteroscopy despite its high prevalence. Here a system ASSIST‐U is proposed to create realistic ureteroscopy images and videos solely using preoperative computerized tomography (CT) images to address these unmet needs. A 3D UNet model is trained to automatically segment CT images and construct 3D surfaces. These surfaces are then skeletonized for rendering. Finally, a style transfer model is trained using contrastive unpaired translation (CUT) to synthesize realistic ureteroscopy images. Cross validation on the CT segmentation model achieved a Dice score of 0.853 ± 0.084. CUT style transfer produced visually plausible images; the kernel inception distance to real ureteroscopy images was reduced from 0.198 (rendered) to 0.089 (synthesized). The entire pipeline from CT to synthesized ureteroscopy is also qualitatively demonstrated. The proposed ASSIST‐U system shows promise for aiding surgeons in the visualization of kidney ureteroscopy.

## Graphical abstract

Here a system ASSIST‐U is presented which automatically and successfully generates patient‐specific and realistic ureteroscopy images from preoperative CT scans. The system uses computer vision methods to automatically segment scans and perform style transfer on the rendered visualizations.

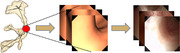

## 1 INTRODUCTION

Endoscopic kidney stone surgery, or ureteroscopy, is a surgical operation that uses a small camera to guide stone removal. Kidney stone removal is a difficult operation due to stone fragments generated during treatment; this is an outcome affected by surgical competency. Expert stone surgeons have a two‐fold higher stone‐free rate compared to less experienced surgeons [1] and analysis of surgical videos shows different patterns in kidney navigation and stone visualization between experts and trainees [2], suggesting an unmet need for surgical training tools. This is particularly important given the high incidence of kidney stone disease (12%) [3] and an almost 30% risk of a repeat procedure after index surgery [4]. Upper tract urothelial carcinoma (UTUC) can be treated similarly by ablating tumours endoscopically, but tumour persistence is common after endoscopic ablation (48–60%) due to missed tumours and incomplete treatment [5]. The high rates of recurrence suggest that the current surgical workflow, where surgeon experience alone dictates surgical approach, may be inadequate. A tool to supplement surgical planning may thus improve outcomes.

**FIGURE 1:**
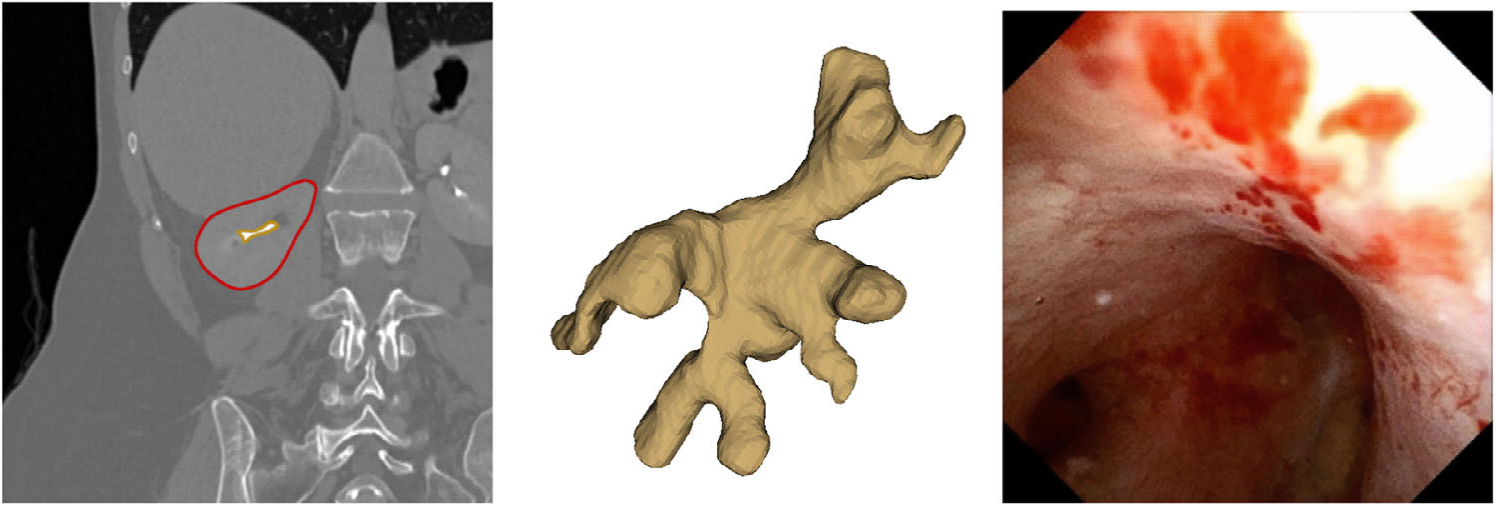
Kidney visualizations. (Left) Computerized Tomography (CT) imaging: Kidney outlined in red, renal pelvis outlined in yellow. (Middle) A rendered mesh of the renal pelvis. (Right) An actual ureteroscopy video. We note the vast differences between the preoperative and intraoperative modalities.

To successfully complete these operations, surgeons must navigate the entire renal collecting system to identify and treat the stones/tumours/fragments. The surgery requires surgeons to create and maintain a mental 3D model of the patient's anatomy solely through preoperative 2D axial computerized tomography (CT) images. This results in a significant mental load, which can negatively impact the success rate of the surgery. This anatomy has a complex shape (Figure 1). The large chamber shown in the central panel is the renal pelvis, or collecting system, and several branching structures (calyces) extend from it. Further complicating the procedure are blood and debris that frequently obscure the camera view, making navigation and stone/tumour/fragment detection a difficult task.

Surgical simulators have improved outcomes and trainee skills in many surgical specialties [6]. Realistic patient‐based simulators could improve not only surgical training but also preoperative planning. However, there are currently no accurate virtual models that enable effective simulators for endoscopic kidney stone or tumour surgery [7]. Thus, there is an unmet need for automated software to develop accurate, patient‐specific models and simulators for preoperative planning and visualization for ureteroscopy.

**FIGURE 2:**
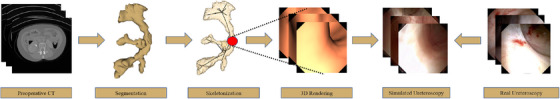
Proposed ASSIST‐U pipeline. We begin by segmenting the pre‐operative computerized tomography (CT) image. Next, we skeletonize the segmentation result. We use points on the skeleton (e.g., the red circle) as camera points for 3D rendering. Finally, we use style transfer to synthesize realistic ureteroscopy images, supervised by the real ureteroscopy data. We note that this figure illustrates the actual results of a subject that has been run through our entire pipeline. Notably, the real ureteroscopy image (far right) suffers from a partial camera occlusion, whereas our synthesized ureteroscopy frame allows better visualization of the local anatomy.

We propose ASSIST‐U (a system for segmentation and image style transfer for ureteroscopy), a preoperative visualization and planning pipeline that leverages preoperative CT to synthesize realistic ureteroscopy videos (Figure 2). The system automatically segments CT scans taken preoperatively (Section 3.2). The segmentation results are used to produce a 3D surface mesh. A skeleton is calculated from this 3D mesh (Section 3.3) and used to generate camera positions to create a 3D rendering of the model (Section 3.4) in VTK [8]. Finally, a style transfer model is applied to simulate realistic ureteroscopy images (Section 3.5). The final results are patient‐specific models and inner anatomy visualizations that have potential for informing surgical planning.

## 2 BACKGROUND

### 2.1 Endoscopic simulation

Surgical simulators allow trainees to practice and develop their skills in a low‐risk environment. Previous simulators developed for ureteroscopic surgical training have facilitated the translation of surgical skills from the training laboratory to the operating room [9]. However, these simulators are often limited in realism and anatomical correctness. The virtual surgical environment is manually animated, and thus it does not react or look like a real intra‐renal tissue or stones. Additionally, it lacks anatomical correctness and does not represent the variations in the possible anatomical configurations of the intra‐renal collecting system [10]. This limits trainee exposure to common intra‐operative situations. Therefore, more realistic and patient‐specific simulations, are needed to improve simulation‐based training [11].

### 2.2 CT Segmentation

Automated segmentation of medical images is a common task, and CT segmentation has been done for a wide variety of organs, such as mandible segmentation for surgical planning [12]. Lin et al. have recently segmented the kidneys and renal mass with 3D UNet [13]. They demonstrated the applicability of the model towards kidney and large tumour segmentation from corticomedullary computed tomography urography (CTU).

### 2.3 Surgical visualization for assisted navigation

There are several systems that aim to provide support for endoscopic surgery through navigational guidance via reconstruction and registration of real‐time video [14, 15]. However, many of these solutions impose equipment demands and/or limit the surgeon's movements. Some require the use of stereoscopes or external sensors, while others generate offline dictionaries for error‐prone lookup [16, 17]. A core issue in these systems is the inability to cope with new and unseen views frequently imposed by the motions typical of endoscopic surgery. A system that can visualize realistic and patient‐specific views could be employed in this navigation task to generate simulations and build navigational maps that allow real‐time scope localization within the kidney during the surgery.

Current methods for navigational and real‐time support during surgery frequently impose some form of cost of deployment as a tradeoff. Our system aims to supplement preoperative imaging purely digitally, with limited computational equipment, and without requiring changes in surgeon workflow during surgery. Importantly, no visualization systems have yet been applied to ureteroscopy, and a specialized solution dealing with the challenges specific to ureteroscopy does not yet exist.

## 3 METHODS

### 3.1 Dataset

#### 3.1.1 Renal pelvis CT dataset

The scans obtained for this study were delayed‐phase CT scans, which are a type of contrast CT administered 6‐15 min after the injection of contrast material. Delayed‐phase CT is used for this study because the contrast material for imaging is most likely in the renal pelvis during this time period, making the targeted renal pelvis brighter and easier to segment.

A total of 17 CT scans were obtained during pre‐operation or return visits via Siemens CT scanners. Of the 17 subjects, 12 were diagnosed with upper tract urothelial carcinoma, three were diagnosed with kidney stones, and two were healthy. The CT protocol involved delayed contrast imaging and the reconstruction of 5 mm slices from acquired helical raw CT scan data (obtained at 0.5 mm slices and 0.2 mm intervals via Siemens CT scanners). The CT scans were manually labeled by a graduate student under the supervision of an experienced urologist using ITK‐SNAP's Active Contour tool (itksnap.org).

#### 3.1.2 Ureteroscopy dataset

Our ureteroscopy dataset consisted of 31 different videos from 21 patients (including three patients overlapping with the CT dataset), with video lengths ranging from 5 to 178 s. These videos were obtained during operations including exploratory surgery, kidney stone removal, and tumour ablation. Videos were sampled at three frames per second (FPS), and complete occlusions by foreign bodies or debris were manually removed. Frames with stones and tumours were also cropped out for style transfer. This resulted in 12,221 images.

### 3.2 CT segmentation

For the automated segmentation of the renal collecting system, we begin by extracting the entire kidney from the CT images using a 3D UNet model. We then isolate the collecting system from the kidneys in post‐processing. We chose not to directly segment the collecting system because it is very small compared to the whole image, creating a significant imbalance of positive and negative labels and making the model prone to noise.

For the preprocessing, the CT scans are first resampled to 256×256×256 voxels to reduce the variability in the CNN input. This also helps reduce the effect of highly anisotropic CT scans, as many of the scans in our dataset have a high in‐plane and low out‐of‐plane resolution (e.g., $0.8\times 0.8\times 5\;{\mathrm{mm}}^{3}$). After resampling, the image resolution ranged from $1.179\times 1.179\times 1.113\;{\mathrm{mm}}^{3}$ to $1.895\times 1.895\times 2.002\;{\mathrm{mm}}^{3}$. The intensity of the scans is then clipped to the [−256,512] HU range and normalized to the [0, 1] range. These values were determined empirically. Next, a series of augmentations are applied to enhance training performance, including random intensity shifts between ±0.026, cropping 16 samples of random 128×128×128 voxels regions with the center of the sampled regions having a balanced foreground–background ratio, random affine transformations with a possibility of rotation between $\pm 30^{\circ }$ and scaling between ±10% in all axes, and a Gaussian smoothing with random σ between 0.5 and 1.5. We use MONAI (https://monai.io) to implement our segmentation model.

The main model is a 3D UNet [18], a classic model widely used for medical image segmentation. The input of the UNet model is a patch of 128×128×128 voxels, as the whole image is too big for our computational resources. For prediction, we use sliding window inference with an overlap ratio of 0.5 and we take the mean of overlapping predictions for the final segmentation result. The model output is a segmentation of the entire kidneys. We ran a sixfold cross validation experiment, with 11 train/3 validation/3 testing subjects per fold.

In post‐processing, first, the kidney segmentation is dilated by a 5×9×9 vox structuring element, ensuring the segmentation captures the whole collecting system. Then, we mask the original CT scan using this dilated kidney segmentation. Finally, we perform a three‐class Otsu [19] thresholding and select the highest intensity label to isolate the collecting system, which is generally highlighted in delayed‐phase CT. This roughly separates the collecting system, kidney, and background into three classes.

### 3.3 Skeletonization

An important part of the rendering pipeline is the camera positioning within the model such that the entire branching structure of the renal collecting system is well represented in the resulting visualization. In ASSIST‐U, we achieve this by performing skeleton extraction^1^ using a method from the computer graphics literature [20]. The algorithm propagates a wave across the surface mesh and records the steps to reach each vertex. Vertices reached at the same step are considered a ring which is then contracted to its center. The step size alters how many vertices are collapsed at each step and the wave count can be used to improve accuracy through averaging.

### 3.4 3D rendering

The binary segmentation of the renal pelvis was converted into a surface mesh using the marching cubes algorithm [21], with a Gaussian smoothing kernel of σ=0.8vox, as implemented in ITK‐SNAP. Three renderers were investigated for creating the surface mesh into images.

1. 3D Slicer's Endoscopy module^2^ was used with the default settings, as a baseline. These are set at 0% ambient, 100% diffuse, and 0% specular lighting. We note that 3D Slicer also renders the triangle mesh edges in a few preset contrasting colors, as can be seen in the top‐left panel of Figure 5. We refer to this model as baseline in this paper.
2. By default, 3D Slicer uses global diffuse lighting; this casts prominent shadows, hiding some of the mesh edges in the rendering. This results in information loss and an extra challenge in the input that the style transfer model must compensate for. We specified 100% ambient lighting and 30% diffuse lighting as an attempt to address this. We refer to this model as CustomSurface.
3. In real ureteroscopy, the only lighting source is always located right next to the camera. To approximate this appearance, we created a standalone Python tool using the Visualization Toolkit (VTK 9.2) [8] rendering libraries with customized settings for lighting. We used 10% specular reflectivity and 50% diffuse lighting with a light source behind the camera to imitate ureteroscopy conditions. Ambient lighting was set to 0%. We did not render the triangle mesh edges in this configuration. We refer to this model as CustomSurfaceAndLight.

For each rendering model, camera trajectories were generated by sampling along the skeleton points, to produce 10,000 rendered images from two manually segmented kidneys for the training set for the style transfer model.

### 3.5 Style transfer

We next train a model that translates images from our 3D rendered solid, textureless “virtual endoscopy” domain (Section 3.4) to the domain of realistic ureteroscopy images. Due to the unpaired nature of our current dataset, we chose CUT [22], a popular unpaired style transfer model, which adopts a patchwise approach to image‐to‐image translation. We trained CUT as described in the original implementation.

## 4 RESULTS

### 4.1 CT segmentation results

**FIGURE 3:**
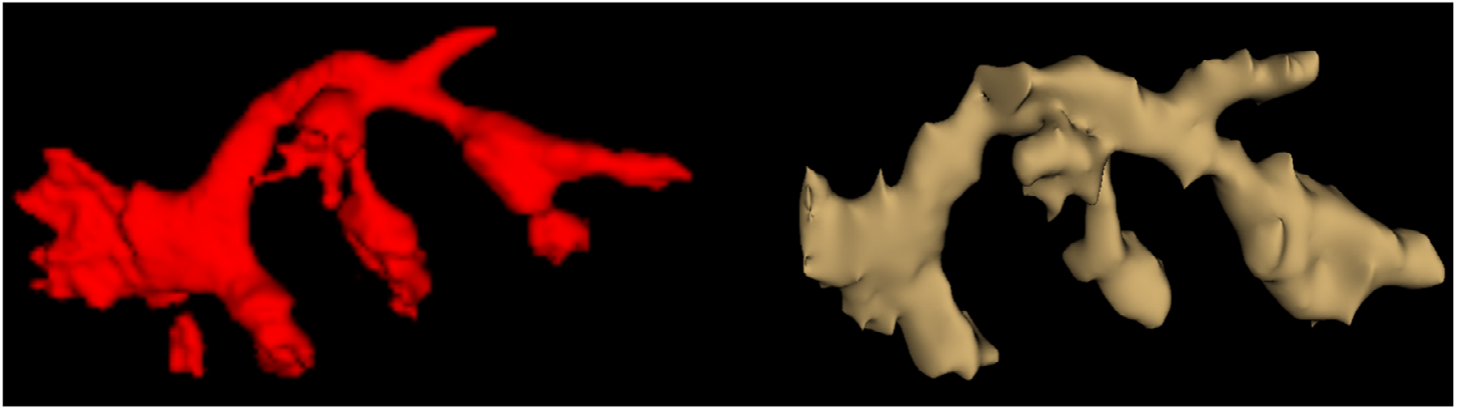
Segmentation results. (Left) Automatic segmentation. (Right) Manual segmentation. We note the overall similarity of the surfaces and, importantly, of the branching structure.

A sixfold cross validation on the UNet model was performed, resulting in an average Dice score of 0.842 ± 0.139 for the entire kidneys, and an average Dice score of 0.853 ± 0.084 for the extracted collecting system. A qualitative example of segmentation results is shown in Figure 3. Visual inspection of the segmentation results suggests that we are able to generate 3D surface meshes that preserve the branching and continuous structure of the renal pelvis, which are important for the subsequent steps of ASSIST‐U. Training was completed on a GTX 1080 GPU in ≈3 h for a single fold. Inference time on an i7‐7820 CPU was on average 90 s per CT volume.

### 4.2 Skeletonization results

The two main parameters of the skeletonization algorithm we use are the step size and wave count. The step size increases the number of rings collapsed at each iteration, whereas the wave count increases the number of waves to achieve a mean value at the cost of introducing noise.

**FIGURE 4:**
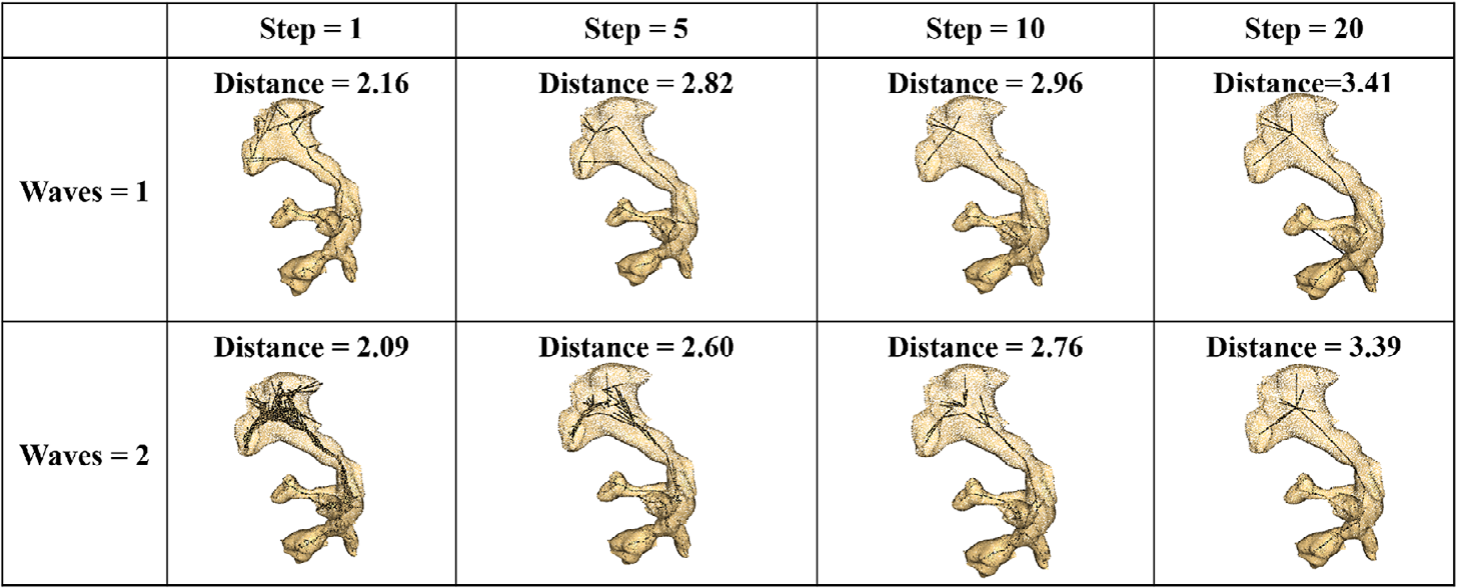
Skeletonization results for different step sizes and number of waves. We note that increasing the step size yields more sparse skeletons and increases the distance metric. Increasing the number of waves improves the fit of the skeleton, reducing the distance metric.

To quantify the effects of the skeletonization parameters, we measure the average distance from each point on the uniformly sampled kidney mesh to the skeleton. A lower distance suggests better coverage of the surface points by the skeleton, which offers a tradeoff against the sparsity of the skeleton. The mesh was sampled at 5000 points for the purposes of this distance computation. These distances as well as qualitative results are reported in Figure 4. As the step size increases, the skeleton becomes more sparse. Increasing the wave count yields a more noisy skeleton but improves the fit and thus reduces the distance; an example can be seen in the lower right panel of Figure 4, where using two waves instead of one fixes the issue of a skeletal point lying outside of the input surface. We selected a step size of five and a wavelength of two for our study. We found that these settings provide a sufficient amount of points in the renal pelvis without drawing too many segments at the upper or lower calyces. The algorithm had a computation time that was under 1 s, proving to be a cost‐effective way to generate a skeleton. Successful skeleton generation provides camera coordinates for 3D rendering of our surface mesh.

### 4.3 3D rendering results

**FIGURE 5:**
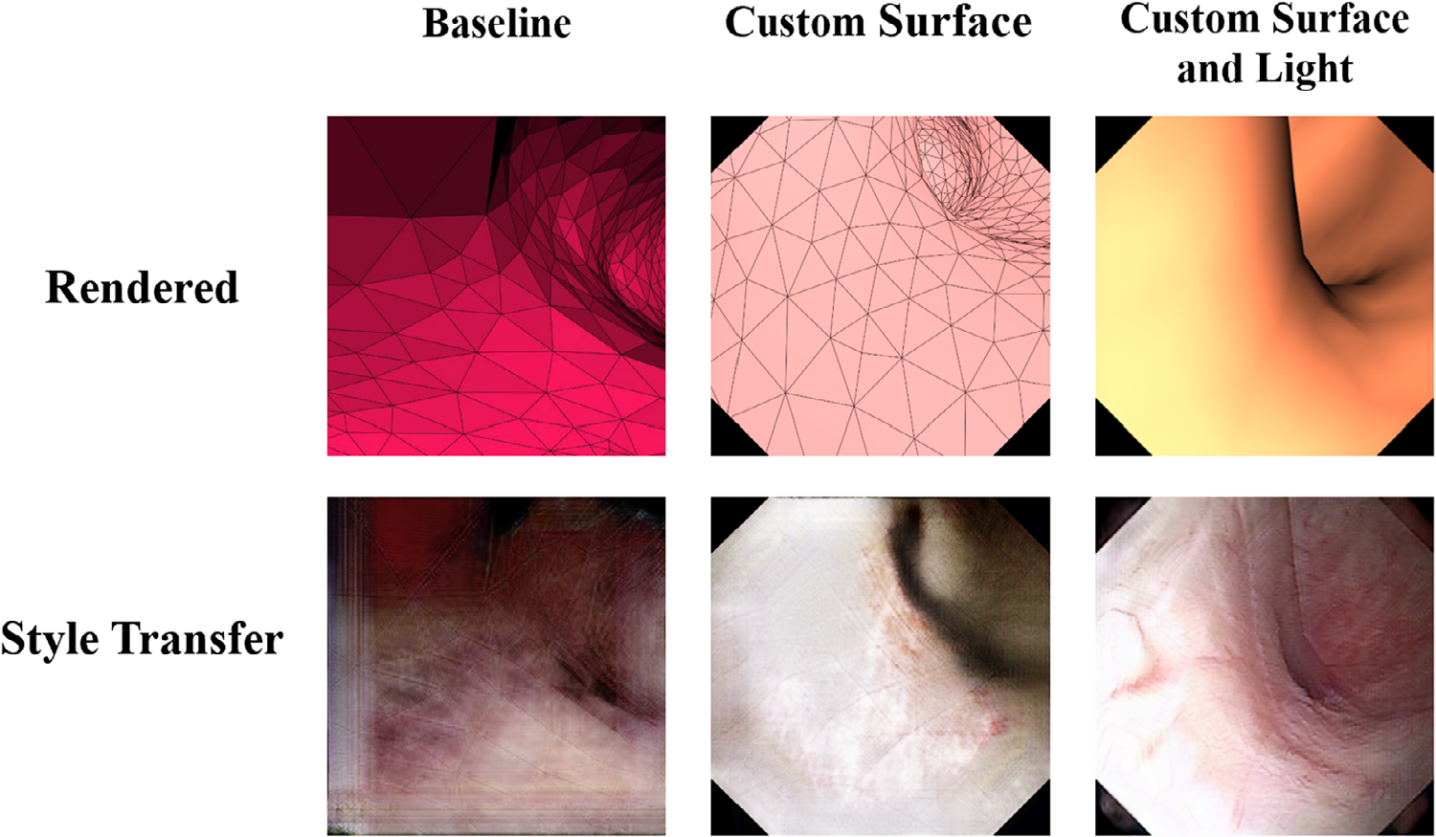
Lighting settings for 3D rendering. (Left) The default lighting parameters for 3D rendering yields artificially dark regions, which can be problematic for style transfer. (Center) 3D rendering with the custom lighting substantially improves the visibility of the rendered image as well as the performance of the style transfer model. (Right) The custom implementation with adjusted light source and surface properties improves the resultant image quality.

Points are sampled along the skeleton edges to obtain camera positions and to render in 3D Slicer. We evaluated the Frechet Inception Distance^3^ [23] and Kernel Inception Distance [24] from the rendered images to the real ureteroscopy images, using the three rendering models. These results are shown in the left panel of Table 1, as well as the left column of Figure 5. Although these metrics do not suggest a clear distinction between the two configurations within 3D Slicer (Baseline vs CustomSurface), we observe that the custom settings improve the results visually. We further observe that the camera position and lighting settings we used in the CustomSurfaceAndLight pipeline substantially improve both these quantitative metrics and the performance of the subsequent style transfer step.

**TABLE 1:** Quantitative evaluation. (Left) Rendering results. (Right) Style transfer results. The Frechet Inception Distance (FID) and Kernel Inception Distance (KID) are evaluated against real ureteroscopy video frames.

	**Baseline**	**CustomSurface**	**CustomSurfaceAndLight**	**Synthetic (Baseline)**	**CustomSurface**	**CustomSurfaceAndLight**
FID	352.698	357.657	**334.211**	237.339	213.512	**186.769**
KID	0.311	0.309	**0.198**	0.199	0.216	**0.089**

### 4.4 Style transfer results

**FIGURE 6:**
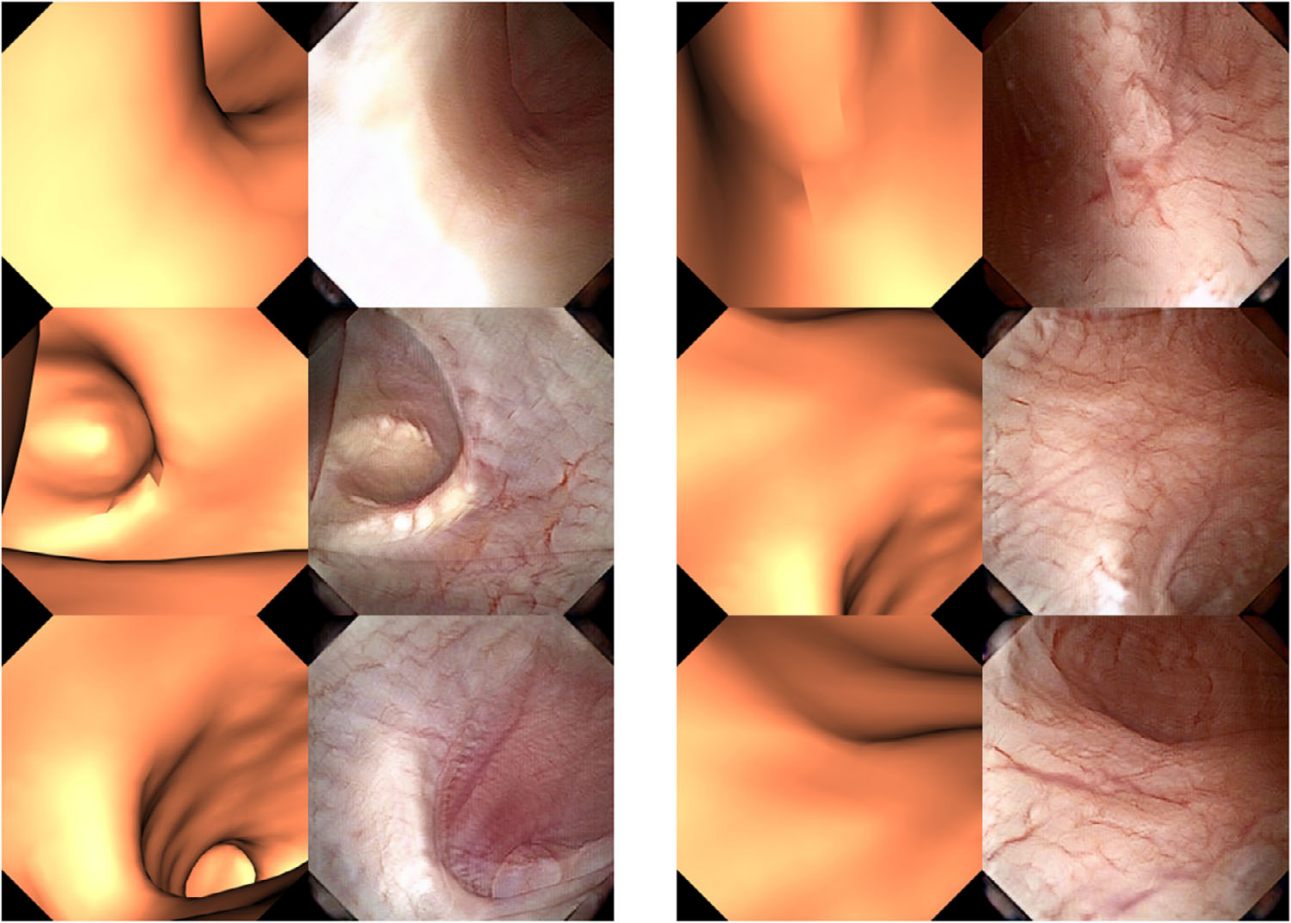
Sample ureteroscopy images generated using our style transfer model. Each pair shows a rendering result with the CustomLightPosition model and the corresponding synthetic style transfer result.

We also evaluated the style transfer results visually and quantitatively. Figure 5 shows the style transfer results for each of the three different 3D rendering settings; it is easy to observe that the custom rendering settings substantially improve the style transfer performance, with the custom light position providing the best results. Additional style transfer outputs for the CustomSurfaceAndLight model are provided in Figure 6.

We again applied the Frechet inception distance (FID) [23] and Kernel inception distance to compare our generated images to real ureteroscopy images (Table 1, right panel). Both metrics are substantially improved by using style transfer compared to direct 3D rendering for each of the renderers, as expected. However, the CustomLightPosition model substantially outperforms the other models.

One of the most difficult issues with using CUT as the style transfer model was the large size and slow training. 200 epochs of training would take ≈60 h to complete depending on the size of the data and models on an RTX 2080ti. However, once trained, the inference time on an i7‐9700KF CPU was on average 49 seconds for 100 images, and only 8 s for 100 images on an RTX 2080ti GPU.

### 4.5 Overall ASSIST‐U pipeline deployment

In previous subsections, we evaluated each component of the ASSIST‐U pipeline individually. Figure 2 shows a qualitative evaluation of the entire ASSIST‐U system for a subject that has been processed through the entire pipeline. The CT image was automatically segmented with the 3D UNet model. The skeletonization algorithm was run with a step size of ten and a wave count of one. Camera positions along the skeleton were used to create 3D rendered images; an example camera position is illustrated in Figure 2 with a red circle. We use the CustomSurfaceAndLight model for rendering. Finally, the style transfer model was used to generate simulated ureteroscopy images. We compared the simulated images with real ureteroscopy images from comparable locations, as illustrated in Figure 2. We note that the overall appearance of the simulated and real ureteroscopy images is similar; additionally, while the real ureteroscopy image suffers from a partial occlusion, the simulated image allows clear visualization of the tissue. This shows the feasibility of using our entire ASSIST‐U pipeline to generate simulated ureteroscopy images from pre‐operative CT images.

As mentioned above, while the training of the models can be computationally expensive, the inference time for new patients is quite short. The models do not need to be retrained per patient. Since the CT segmentation inference can be run pre‐operatively, the only overhead to the workflow is the inference time for style transfer. This is, as indicated above, about half a second per frame on a GPU, so we do not expect workflow disruptions.

## 5 DISCUSSION

In this paper, we propose ASSIST‐U, a system for realistic visualization of ureteroscopy from preoperative CT images. The model segmented the collecting system with a mean Dice score of 0.853. A skeletonization method was used to generate camera trajectories inside the collecting system for 3D rendering. We explored three different rendering settings for visualization. Finally, a style transfer model was trained to transfer between the rendered and synthetic ureteroscopy domains.

The achieved Dice score demonstrates that our model is capable of capturing the entire continuous structure of the renal collecting system. This is sufficient for generating visualizations for clinical usage, since a skeleton with the correct branching structure is more critical than precise surface placement for our purposes. This is especially true since the surface is likely to deform during ureteroscopy. Style transfer performance, while it can be improved, clearly demonstrates that we can achieve much more realistic simulations than the rendered images. Combined with the patient‐specific nature of our simulation, this increased realism is expected to benefit surgeon training and surgical planning compared to using rendered images from pre‐set anatomy.

Our comparison of the three rendering models shows that more suitable rendering settings, such as realistic light source position and surface properties, can vastly improve the simulation results. We also note that the poor visibility conditions in the Baseline and CustomSurface models make it necessary to also render the triangle edges to capture depth information, which do not fully disappear during style transfer. Without triangle edges, depth perception becomes more difficult, and the global lighting results in misleading shadows. Instead, the custom pipeline we implemented allows the light reflectance to be used to encode this information, without the artifacts caused by the triangle edges during style transfer.

A limitation of this study was the difficulty of curating a large, high‐quality dataset. Although our CT dataset was a good representation of the heterogeneity of CT scans in our patient population (e.g., patients with only one functional kidney, patients with metallic implants, CT scans acquired from older machine models, etc.), the lack of quantity in any one category has created challenges. While our results indicate we achieved a good balance, the model would no doubt benefit from a much larger training set.

A benefit of applying a generative style transfer model is that camera angle and surface deformations would not impact the ability of the model to create realistic images. However, we note that if CT segmentation produces a model with disconnected regions, this would result in problems during skeletonization and rendering. The camera renders would likely fail to show the disconnected region. Additionally, the model does not have temporal regularization, so further work is needed to make the compiled video more consistent.

We note that the style transfer task would greatly benefit from paired rendered and in vivo ureteroscopy images that would allow the use of more advanced models and more precise numerical analysis of task performance. In future work, we will perform tissue reconstruction from endoscopic video and register it for CT scans to enable such an analysis. Registering in vivo uterescopy video would allow the determination of accurate camera positioning for paired datasets.

The intermediate steps of our pipeline can also be used for other purposes outside the main ASSIST‐U workflow. For example, in addition to providing individual camera positions, the entire skeleton can also be used to allow a better understanding of the branching structure of the collecting system. Additionally, the modularity of our system makes it easy to implement new features for each component in future work, as well as allowing manual intervention at each step in failure cases. This makes ASSIST‐U very straightforward to adapt to new modalities or even new domains.

In the surgical workflow, we envision ASSIST‐U to be utilized preoperatively, allowing clinicians the ability to interact with a patient collecting system model days beforehand to better understand the operating site. The structure of this model would be correct and textured realistically according to the preoperative CT imaging. This would potentially allow a surgeon to understand how each region would look from different camera angles, which may not be possible during the actual operation. As a simulator, it could also be used as a way to allow notable cases to be presented for trainees to experience in future virtual reality environments.

## 6 CONCLUSION

Our ASSIST‐U system successfully generates patient‐specific and realistic ureteroscopy images without any requirements for external hardware or manual expert labeling. These images can be used for preoperative visualization and surgical planning, as well as surgical training. The system could reduce the cognitive load of the surgeon during surgery by showing patient‐specific operating site visualizations, and thus potentially lowering the need for mental mapping, and could help reduce the amount of stones, residual stone fragments or tumours in unexplored branches of the collecting system. Additionally, this system provides a step towards the development of a realistic surgical training tool as well as a surgical navigation system.

## AUTHOR CONTRIBUTIONS

**Daiwei Lu**: Conceptualization; data curation; formal analysis; investigation; methodology; software; validation; visualization; writing—original draft; writing—review and editing. **Yifan Wu**: Formal analysis; investigation; methodology; software. **Ayberk Acar**: Investigation; methodology; writing—review and editing. **Xing Yao**: Formal analysis; investigation; methodology; software. **Jie Ying Wu**: Project administration; supervision; writing—review and editing. **Nicholas Kavoussi**: Data curation; funding acquisition; project administration; supervision; writing—review and editing. **Ipek Oguz**: Funding acquisition; project administration; supervision; writing—review and editing.

## CONFLICT OF INTEREST STATEMENT

The authors declare no conflicts of interest.

## Data Availability

The code will be made publicly available (http://github.com/MedICL‐VU). The data is currently not publicly available.

## Footnotes

1
https://github.com/navis‐org/skeletor

2
https://slicer.readthedocs.io/en/latest/user_guide/modules/endoscopy.html

3
https://github.com/mseitzer/pytorch‐fid
